# Echinacoside Alleviates Cisplatin-Induced Acute Kidney Injury by Regulating Bap1 to Inhibit Ferroptosis

**DOI:** 10.3390/biomedicines14092127

**Published:** 2026-09-20

**Authors:** Lijia Chen, Mingjie Zhang, Miao Chi, Zehua Ye, Bojun Li, Songyuan Yang, Miao Zhang, Wei Dong, Haoyong Li, Xiangjun Zhou

**Affiliations:** 1 Department of Urology, Renmin Hospital of Wuhan University, No. 238 Jiefang Road, Wuchang District, Wuhan 430060, China; lijiachen.md@whu.edu.cn (L.C.); 2025283020115@whu.edu.cn (M.Z.); 2021305233113@whu.edu.cn (M.C.); yezehua0704@163.com (Z.Y.); libojun@whu.edu.cn (B.L.); ysy097019@163.com (S.Y.); 2020305232025@whu.edu.cn (M.Z.); 2 Department of Urology, Union Hospital, Tongji Medical College, Huazhong University of Science and Technology, Wuhan 430022, China

**Keywords:** echinacoside, acute kidney injury, ferroptosis, Bap1, cisplatin, HK-2

## Abstract

**Background/Objectives:** Cisplatin-induced acute kidney injury (AKI) is a major adverse complication restricting cisplatin-based chemotherapy. Ferroptosis contributes substantially to proximal tubular epithelial injury during AKI. This work aimed to explore the renoprotective effect of echinacoside (ECH) and its underlying molecular mechanism against cisplatin-provoked AKI. **Methods:** Eight-week-old male C57BL/6 cisplatin-AKI mice models and HK-2 tubular epithelial cells were used for in vivo and in vitro experiments. Public single-cell transcriptome data and 4D-DIA proteomics were applied to screen key ferroptosis-associated molecules. Functional validation, Western blotting, and molecular docking simulations were performed to characterize the regulatory axis of ECH. **Results:** ECH ameliorated renal dysfunction, inflammation, and tubular damage in cisplatin-induced AKI. Omics analyses nominated Bap1 as a critical ferroptosis-related differential protein. ECH restored the Slc7a11/GPX4 pathway to restrain lipid peroxidation and mitochondrial ferroptotic damage. Molecular docking predicted a potential interaction between ECH and Bap1. ECH downregulated Bap1 protein expression, and Bap1 overexpression largely abrogated ECH-mediated anti-ferroptosis and renoprotective effects. **Conclusions:** ECH mitigates cisplatin-triggered AKI, which is associated with Bap1 downregulation, Slc7a11/GPX4 axis restoration, and ferroptosis suppression. The ECH-Bap1-ferroptosis regulatory relationship offers a candidate therapeutic target and natural agent for the preventive treatment of cisplatin-related nephrotoxicity.

## 1. Introduction

Cisplatin is a first-line chemotherapeutic agent widely applied in the clinical treatment of multiple solid tumors, including testicular, ovarian, bladder, and lung cancers [1,2]. Nevertheless, severe dose-dependent acute kidney injury (AKI) represents its most prominent adverse effect, which drastically restricts clinical dosage and leads to increased hospitalization rates, renal dysfunction, and even progression to chronic kidney disease (CKD) in long-term survivors [3,4]. At present, supportive treatments such as hydration can only partially reduce cisplatin nephrotoxicity, while specific pharmacological interventions targeting tubular injury are still lacking [5,6,7]. Therefore, it is urgent to identify safe and effective renoprotective agents to relieve cisplatin-triggered AKI and reveal their precise molecular mechanisms.

Ferroptosis, a distinctive iron-dependent programmed cell death characterized by massive lipid peroxidation, glutathione depletion, and mitochondrial structural destruction, has been proven to act as a central pathological event driving renal tubular epithelial damage during cisplatin-induced AKI [8,9,10]. The Slc7a11-GPX4 axis serves as the core antioxidant defense system suppressing ferroptosis: Slc7a11 mediates cystine uptake to maintain intracellular glutathione (GSH) homeostasis, whereas GPX4 eliminates toxic lipid peroxides to block the ferroptotic cascade [11,12,13]. Accumulating evidence demonstrates that cisplatin significantly downregulates Slc7a11 and GPX4 expression in proximal tubular cells, resulting in disrupted redox balance, lipid ROS accumulation, and irreversible tubular damage [14,15,16]. Targeting the upstream regulatory molecules that control Slc7a11/GPX4 transcription may provide a novel strategy to inhibit ferroptosis and alleviate cisplatin nephropathy.

Bap1 acts as an epigenetic transcriptional repressor that suppresses the expression of multiple antioxidant genes [17,18]. Previous studies have reported that the PR-DUB complex, in which Bap1 serves as the catalytic subunit, represses Slc7a11 transcription by removing H2AK119ub histone modifications at the Slc7a11 promoter, thereby accelerating ferroptosis under oxidative stress [19,20]. Upregulated Bap1 expression is observed in renal tissues of cisplatin-challenged mice, whereas inhibition of Bap1 effectively restores Slc7a11/GPX4 activity and mitigates ferroptotic injury [21,22]. However, no natural small-molecule compound has been reported to modulate Bap1 and relieve cisplatin-induced AKI by regulating Bap1-mediated ferroptosis signaling.

Echinacoside (ECH), a bioactive flavonoid monomer isolated from traditional Chinese medicinal herbs, possesses extensive pharmacological activities, including anti-inflammatory, anti-oxidative, and organ-protective effects [23]. Previous studies have validated that ECH exerts protective functions in multiple organ injury models by scavenging reactive oxygen species and restraining inflammatory cascades [24,25,26,27]. Nevertheless, whether ECH can ameliorate cisplatin-induced AKI, and whether its renoprotective effect is associated with Bap1 and ferroptosis, remain unexplored.

Recently, Fan et al. (2025) reported that ECH alleviates cisplatin-induced AKI by activating the NRF2/HO-1 antioxidant cascade, which mainly counteracts cisplatin-triggered oxidative stress and inflammatory responses [25]. However, their work did not explore the regulatory link between ECH and ferroptosis, nor identify upstream molecular targets governing the Slc7a11/GPX4 axis. Distinct from the NRF2-centered mechanism described previously, the present study focuses on the epigenetic repressor Bap1 and its downstream ferroptosis signaling. Through multi-omics screening, molecular simulation, and genetic rescue assays, we identified a novel Bap1-dependent regulatory relationship associated with Slc7a11/GPX4 restoration and tubular ferroptosis suppression, which represents a unique incremental innovation compared with the published NRF2-related mechanism of ECH.

This study constructed in vivo and in vitro cisplatin-induced AKI models to assess ECH’s renoprotective capacity. Multi-omics screening, cell functional assays, molecular simulation, and Bap1 rescue experiments were integrated to show that ECH downregulates Bap1 to restore Slc7a11/GPX4 signaling and block ferroptosis, thereby providing novel theoretical evidence and a candidate agent for treating cisplatin-related renal injury.

## 2. Materials and Methods

### 2.1. In Vivo Animal Experiments

All animal experiments complied with the NIH laboratory animal guidelines and ARRIVE 2.0 guidelines and were approved by the Animal Ethics Committee of Renmin Hospital of Wuhan University. Eight-week-old male C57BL/6 mice obtained from Wuhan University were housed under SPF conditions at 22 ± 2 °C and 55 ± 5% humidity, with a 12 h light/12 h dark cycle and free access to standard chow and drinking water. A single individual mouse was defined as the experimental unit in this in vivo study.

Mice were randomly allocated to experimental groups using a simple random-number-generation method to minimize cage location-related confounding effects. Investigators performing histological scoring, biochemical quantification, and image analysis were blinded to group allocation; blinding was not feasible during drug administration and tissue harvesting procedures due to practical experimental constraints. Predefined inclusion–exclusion criteria for animals were not established prior to experimentation. No animals or data points were excluded from the final analysis in this study. Sample sizes per group were selected based on previously published cisplatin AKI pre-clinical literature and our preliminary in-house pilot observations; no formal a priori power analysis was performed. Animals were subdivided for different experimental endpoints: *n* = 3 mice per group were used for quantitative analyses, including serum biochemistry, histological scoring, and transmission electron microscopy assessment, and the remaining animals were used for transcriptome sequencing and tissue specimen preservation. Humane endpoints were predefined, including >20% body weight loss, persistent lethargy, hunched posture, or impaired feeding and drinking behavior; animals meeting these criteria would be humanely euthanized immediately. No animals reached these humane endpoint criteria during this study.

For cisplatin-induced AKI, cisplatin (1 mg/mL in sterile PBS) was intraperitoneally injected once at 25 mg/kg, and mice were sacrificed 48 h later by cervical dislocation under isoflurane anesthesia. Low-dose (10 mg/kg) or high-dose (30 mg/kg) ECH dissolved in sterile saline was administered via daily intraperitoneal injection for 7 consecutive days, starting 5 days prior to cisplatin injection and lasting until sacrifice. Normal control mice received an equal volume of 0.9% saline on the same schedule. At sacrifice, peripheral blood and whole-kidney tissues were collected for serum biochemistry, histological staining, transmission electron microscopy, transcriptome sequencing, total protein extraction, and other molecular analyses.

### 2.2. Cell Culture and In Vitro Treatments

Human renal tubular epithelial HK-2 cells (Catalog No. QS-H025, KEYCELL Biotechnology, Wuhan, China) were cultured in DMEM/F12 (11320033, Gibco, Thermo Fisher Scientific, Grand Island, NY, USA) complete medium supplemented with 10% fetal bovine serum (AUS-01S-02, Cell-BOX (HK) Biological Products Trading Co., Ltd., Changsha, China) and 1% penicillin–streptomycin–amphotericin B mixed solution (BL142A, Biosharp, Beijing, China), and maintained at 37 °C in a humidified incubator containing 5% CO_2_. To establish the in vitro cisplatin-induced tubular injury model, HK-2 cells were exposed to 20 μM cisplatin (S1166, Selleck Chemicals, Shanghai, China) for 24 h. Serial concentrations of ECH (HY-N0020, MedChemExpress, Monmouth Junction, NJ, USA) were pre-incubated with cells for 2 h before cisplatin stimulation to investigate the renoprotective activity of ECH. For the rescue functional assay, HK-2 cells were transfected with a Bap1 overexpression plasmid or an empty control plasmid, followed by co-treatment with 20 μM cisplatin and corresponding ECH concentrations to validate whether Bap1 overexpression could abrogate the protective effect of ECH.

### 2.3. Cell Viability Assay

Cell viability was detected using a CCK-8 kit (C0037, Beyotime, Shanghai, China) according to the manufacturer’s instructions. The absorbance value of each well was measured using a microplate reader, and the survival rate of HK-2 cells treated with single ECH or cisplatin combined with gradient ECH was calculated based on absorbance data.

### 2.4. PI Staining for Cell Death Detection

HK-2 cells were fixed and incubated with PI nuclear staining solution (C1188-100 mL, Beyotime, Shanghai, China). Fluorescence images were captured using an inverted fluorescence microscope (Ts2R, Nikon, Tokyo, Japan) equipped with an Olympus DP74 imaging system (Olympus, Tokyo, Japan). ImageJ software (version 1.54g) was used to quantify the proportion of PI-positive dead cells to assess cell death levels in each group.

### 2.5. C11-BODIPY Staining for Intracellular Lipid Peroxidation

The intracellular lipid peroxidation level was labeled using the C11-BODIPY 581/591 fluorescent probe (MX5211-1MG, Meilunbio, Dalian, China), with the red fluorescence signal representing non-oxidized lipids and the green fluorescence signal representing oxidized lipids. A confocal microscope was used to acquire fluorescence images, and the fluorescence intensity of oxidized lipids was statistically analyzed to reflect the degree of lipid peroxidation.

### 2.6. Biochemical Index Detection

Kidney tissue homogenate and cell lysis supernatant were prepared, respectively, and commercial biochemical detection kits were used to detect the contents of superoxide dismutase, reduced glutathione, oxidized glutathione, malondialdehyde, and ferrous iron in the samples; meanwhile, the ratio of GSH to GSSG was calculated for further analysis of the intracellular redox state.

### 2.7. ELISA Detection of Serum Renal Injury Markers

Serum samples collected from mice were subjected to ELISA using detection kits (ELK Biotechnology, Wuhan, China) to measure the concentrations of serum BUN, creatinine, KIM-1, and NGAL, strictly following the operating specifications provided by the kit manufacturer, to evaluate the degree of renal injury in each group of animals.

### 2.8. Quantitative Real-Time PCR (qRT-PCR)

Total RNA was extracted from mouse kidney tissues and HK-2 cells, respectively, and a reverse transcription reaction was carried out to synthesize complementary DNA. Quantitative real-time PCR was performed to detect the relative mRNA expression of inflammatory factors, including IL-1β, MCP-1, TNF-α, and IL-6, as well as the ferroptosis-related gene Bap1, and the target gene expression levels were normalized to an internal reference gene for comparative analysis between groups.

### 2.9. Western Blot Analysis

Total protein was extracted from renal tissues and HK-2 cells, and equivalent protein samples were separated by SDS-PAGE gel electrophoresis and transferred to PVDF membranes. After membrane blocking, primary antibodies targeting KIM-1 (A24493, ABclonal, Wuhan, China), NGAL (A2092, ABclonal, Wuhan, China), GPX4 (A27995, ABclonal, Wuhan, China), Slc7a11 (A25302, ABclonal, Wuhan, China), Bap1 (A6533SP, ABclonal, Wuhan, China) and the internal reference GAPDH (A19056, ABclonal, Wuhan, China) were incubated overnight at 4 °C, followed by incubation with corresponding secondary antibodies. Protein bands were visualized using a chemiluminescence exposure system, and the gray value of each band was quantified with ImageJ software to calculate the relative protein expression levels of target proteins normalized to GAPDH.

### 2.10. Tissue Staining Assays

Paraffin sections of mouse kidney were prepared for hematoxylin–eosin staining and periodic acid–Schiff staining to observe the overall pathological damage and tubular structural lesions of renal tissues, and paraffin sections were also incubated with a KIM-1 primary antibody for immunohistochemical staining to observe the tissue distribution of the KIM-1 injury marker. Frozen kidney sections were prepared for GPX4 immunofluorescence staining; a GPX4 primary antibody and a fluorescent secondary antibody were used for specific labeling, DAPI was applied for nuclear counterstaining, and a confocal microscope was used to capture images and quantify GPX4 fluorescence intensity in renal tissue.

### 2.11. Transmission Electron Microscopy

Renal tubular tissue blocks from mice were fixed, dehydrated, and embedded to prepare ultrathin sections; mitochondrial morphological changes in renal tubular epithelial cells were observed under a transmission electron microscope, and the aspect ratio of mitochondria was statistically quantified to evaluate mitochondrial structural damage induced by ferroptosis.

### 2.12. Public Single-Cell RNA-Seq Data Analysis

Public single-cell RNA-seq data (GSE220675) of cisplatin-injured mouse kidneys were re-analyzed using R and Seurat v4.3.0. The GSE220675 dataset was derived from mouse kidneys (Mus musculus). Cisplatin-induced AKI was established by a single intraperitoneal injection of 20 mg/kg cisplatin, and kidney tissues were collected on day 3 and day 7 after cisplatin administration. This cisplatin dosing regimen is consistent with the in vivo AKI model applied in our study, supporting cross-dataset comparability. Cells with nFeature_RNA < 200 or >6000 or a mitochondrial gene percentage > 15% were filtered. Datasets were normalized and integrated via SCTransform (Seurat v4.3.0). The top 20 principal components were selected for UMAP dimensional reduction according to the elbow-plot heuristic.

Differentially expressed genes (DEGs) between control and injured proximal tubular cells were identified using FindMarkers (Seurat v4.3.0) with the following thresholds: |log_2_FC| > 0.25 and adjusted *p*-value < 0.05. Cell types were annotated with canonical marker genes. Ferroptosis signature scores were calculated by AddModuleScore (Seurat v4.3.0) using the MSigDB (v7.5.1) ferroptosis gene set. Violin plots visualized the expression of ferroptosis scores and marker genes (ACSL4, TFRC, and ALOX15). DEGs from proximal tubular cells were subjected to KEGG enrichment analysis.

### 2.13. 4D-DIA Quantitative Proteomics

A total of 7374 proteins were identified by 4D-DIA mass spectrometry. Differentially expressed proteins (DEPs) between groups were screened with the following thresholds: |log_2_FC| > 0.25 and FDR < 0.05, yielding 132 DEPs. Global DEPs were subjected to KEGG and GSEA enrichment analyses. Candidate proteins associated with ferroptosis were further filtered by intersecting DEPs with ferroptosis-related gene sets from public databases. Among these candidates, Bap1 was prioritized for further validation based on its expression alteration, reported regulatory role in ferroptosis, and relevance to renal injury.

### 2.14. Molecular Docking

Crystal structures of human Bap1 and SAT1 were retrieved from the PDB database. After removing redundant water and heteroatoms, polar hydrogens were added, and Gasteiger charges were assigned to protein residues. The 3D conformation of ECH was constructed and geometrically optimized via the conjugate gradient algorithm. Semi-flexible docking was performed with AutoDock Vina (v1.2.6), and the grid box was centered on the ligand-binding pocket to fully cover the active cavity. The pose with the lowest binding free energy was selected for visualization. Discovery Studio Visualizer was used to display binding pocket residues, hydrogen bonds, and hydrophobic interactions, and to generate 2D interaction diagrams. SAT1, a well-reported ferroptosis-associated protein also detected in our 4D-DIA proteomic dataset, was employed as a reference ferroptosis-related receptor for predicted docking affinity comparison against Bap1 [28,29]. Of note, SAT1 is not an experimentally confirmed binding target of ECH. Our computational screening was focused on these two proteomics-identified ferroptosis-associated candidates; expanded docking profiling covering more ferroptosis-regulating receptors will be explored in future investigations.

### 2.15. Molecular Dynamics Simulation

The optimal Bap1-ECH docking complex was subjected to a 100 ns all-atom MD simulation using Gromacs 2022. Amber99sb-ildn and GAFF force fields were adopted for the protein and ECH ligand, respectively. The complex was solvated in a cubic SPCE water box, and counterions were added to neutralize the system charge. The PME algorithm handled electrostatic interactions, with a 1.2 nm cutoff for Coulomb and van der Waals forces. Steepest descent minimization with a maximum of 500,000 steps eliminated steric clashes, followed by 100 ps NVT equilibration (298.15 K, V-rescale thermostat) and 100 ps NPT equilibration (1 bar, Parrinello–Rahman barostat). Periodic boundary conditions were applied during the 100 ns production simulation.

Gromacs built-in tools analyzed trajectory data, including RMSD, RMSF, Rg, SASA, and the number of protein–ligand hydrogen bonds. Ligand centroid distance was calculated to evaluate binding pocket stability. gmx_MMPBSA decomposed total binding free energy into individual residue contributions to identify critical interacting residues. Trajectories were visualized with VMD. Notably, only a single 100 ns production-phase MD trajectory with one random seed was performed for the ECH-Bap1 complex in the present study.

### 2.16. Statistical Analysis

GraphPad Prism 8.0 was utilized to conduct all statistical computations. All quantified results were expressed as the mean ± standard deviation. Prior to one-way ANOVA, the normality of the data distribution and homogeneity of variance were assessed. Two-group comparisons were performed using an unpaired *t*-test. For multiple-group comparisons, one-way analysis of variance (ANOVA) was applied followed by Tukey’s multiple comparisons post hoc test. Biological replicate numbers for each experiment are indicated in the corresponding figure legends, with a minimum of three independent biological replicates. Any *p*-value less than 0.05 was judged to represent a statistically significant difference. Explanations of significance symbols are listed in each figure legend.

## 3. Results

### 3.1. ECH Alleviates Cisplatin-Induced Renal Dysfunction and Renal Inflammatory Injury In Vivo

**Figure 1:**
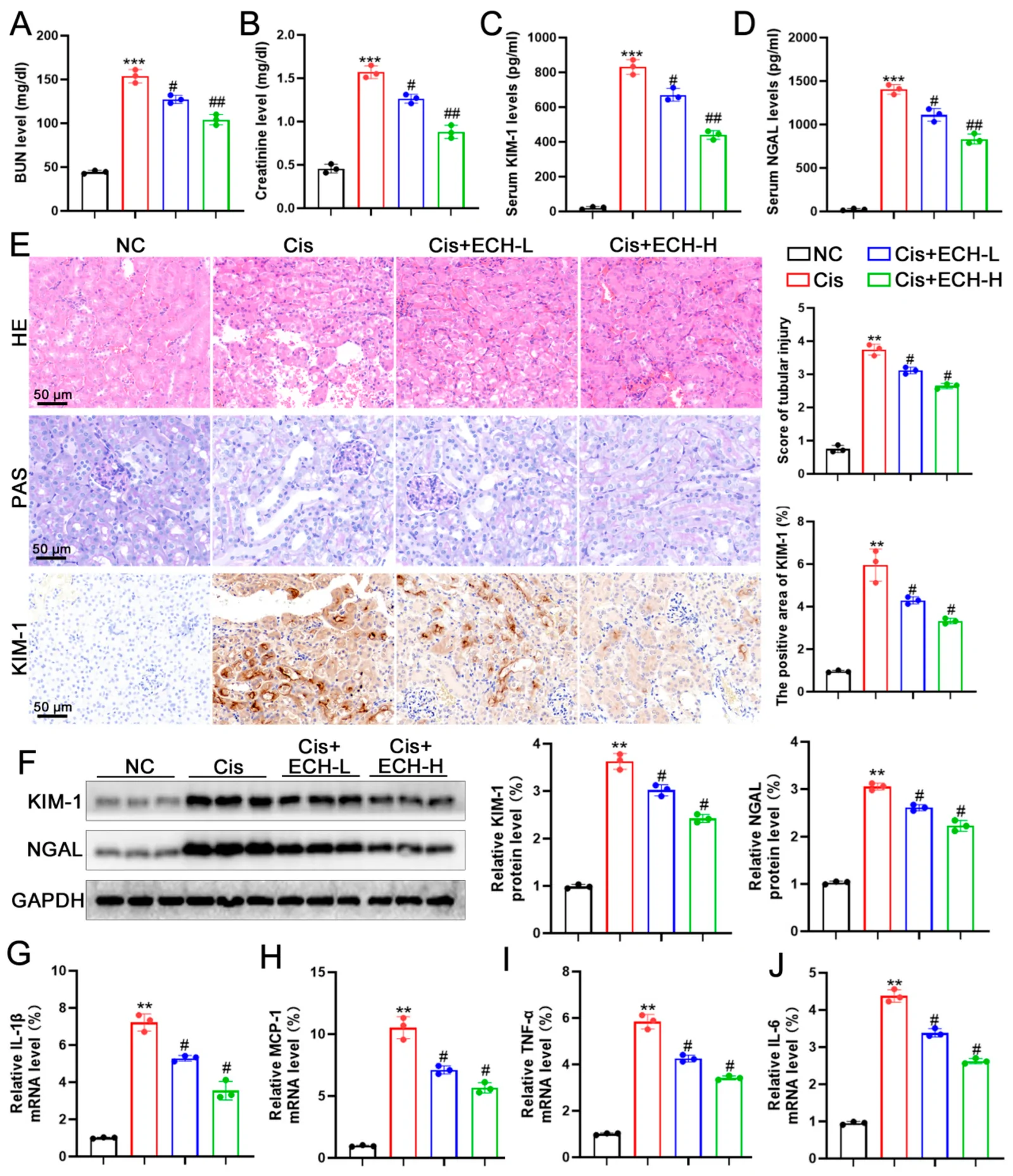
ECH alleviates cisplatin-induced renal injury and inflammation in vivo. (**A**) Serum BUN levels in each group. (**B**) Serum creatinine levels of each group. (**C**) Serum KIM-1 levels detected by ELISA. (**D**) Serum NGAL levels detected by ELISA. (**E**) Representative H&E, PAS and KIM-1 immunohistochemical staining images of kidney tissues and quantitative analysis. (**F**) Western blot analysis and quantitative analysis of KIM-1 and NGAL protein in kidney tissues. (**G**–**J**) Relative mRNA levels of IL-1β, MCP-1, TNF-α and IL-6 in kidney tissues. ** *p* < 0.01, *** *p* < 0.001 vs. control group; ^#^ *p* < 0.05, ^##^ *p* < 0.01 vs. cisplatin group. *n* = 3 mice per group.

To verify the renoprotective effect of ECH in cisplatin-triggered AKI, we first detected serum renal injury indicators. Compared with the normal control (NC) group, serum BUN and creatinine levels were markedly elevated in cisplatin-challenged mice (Figure 1A,B). Meanwhile, ELISA results revealed dramatic increases in renal injury markers KIM-1 and NGAL in serum after cisplatin exposure (Figure 1C,D). Both low-dose (ECH-L) and high-dose (ECH-H) ECH pretreatment significantly reversed the above biochemical abnormalities, with a more prominent effect observed in the high-dose group.

Histopathological staining further validated the renoprotective phenotypes. H&E and PAS staining displayed severe tubular dilatation, brush border loss, and tubular necrosis in the cisplatin group, whereas ECH intervention alleviated tubular structural lesions. Immunohistochemical staining showed abundant KIM-1-positive signals in cisplatin-damaged renal tubules, which were obviously suppressed by ECH administration (Figure 1E). Consistently, Western blot quantification demonstrated that cisplatin induced robust upregulation of KIM-1 and NGAL proteins in kidney tissues, and ECH dose-dependently downregulated these injury proteins (Figure 1F).

qRT-PCR analysis of inflammatory mediators revealed that the mRNA expression of IL-1β, MCP-1, TNF-α, and IL-6 was sharply elevated in cisplatin-treated renal tissues. ECH treatment remarkably reduced the transcription of these pro-inflammatory factors, indicating that ECH mitigated the cisplatin-evoked renal inflammatory response (Figure 1G–J). Collectively, these in vivo data demonstrated that ECH effectively ameliorated cisplatin-induced renal dysfunction, tubular pathological damage, and inflammatory infiltration.

### 3.2. ECH Protects HK-2 Cells Against Cisplatin-Mediated Tubular Epithelial Damage In Vitro

**Figure 2:**
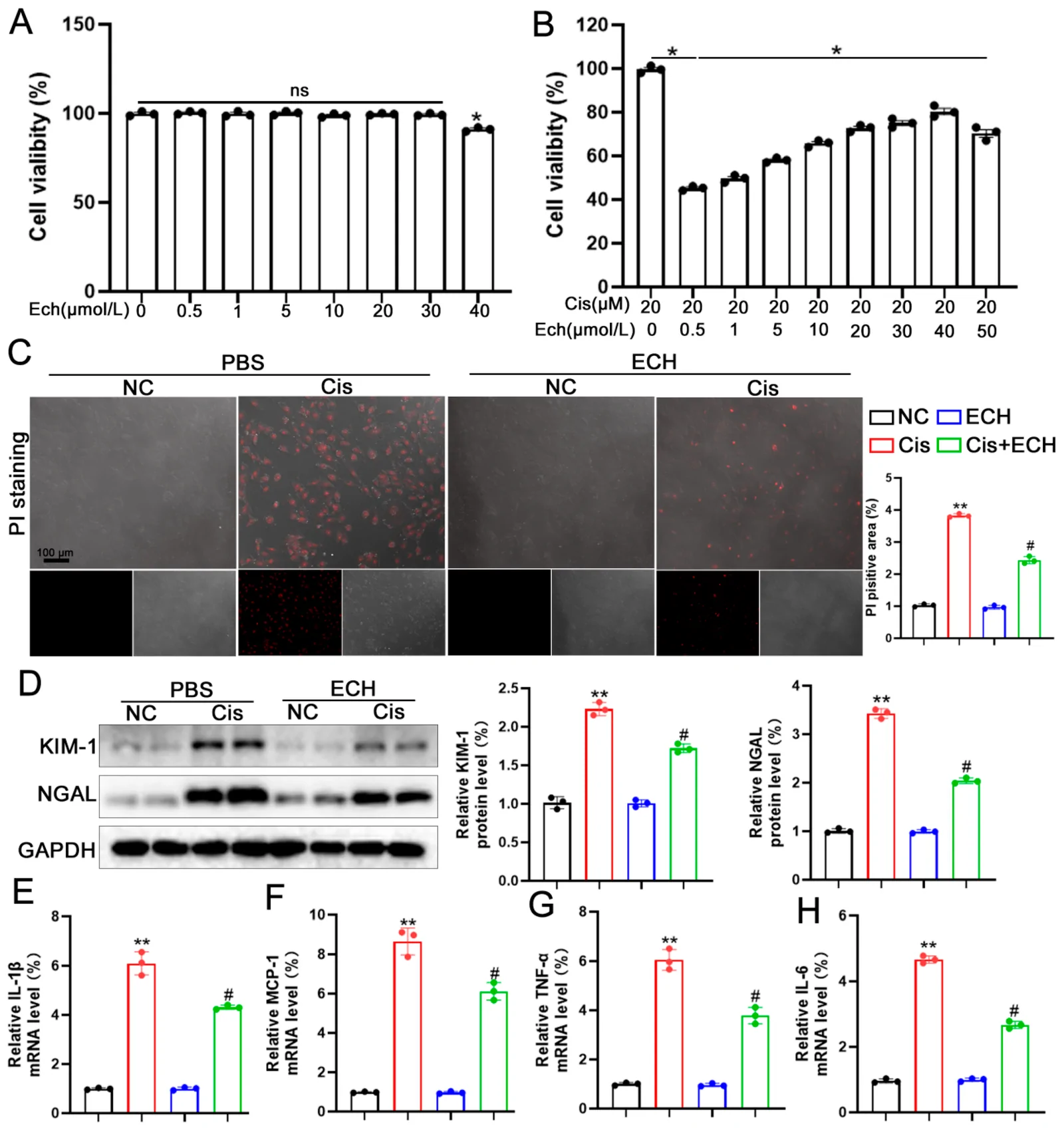
ECH relieves cisplatin-induced cell damage in vitro. (**A**) HK-2 cell viability treated after treatment with different concentrations of ECH. (**B**) HK-2 cell viability under cisplatin stimulation with gradient ECH intervention. (**C**) Representative PI staining images of HK-2 cells and quantitative analysis. (**D**) Western blot and quantitative analysis of KIM-1 and NGAL proteins in HK-2 cells. (**E**–**H**) Relative mRNA levels of IL-1β, MCP-1, TNF-α and IL-6 in HK-2 cells. ns, no significancant; * *p* < 0.05, ** *p* < 0.01 vs. control group; ^#^ *p* < 0.05 vs. cisplatin group.

A CCK-8 assay was performed to exclude the cytotoxicity of ECH alone and evaluate its cytoprotective activity. ECH at concentrations ranging from 0.5 to 30 μmol/L exerted no obvious toxic effects on HK-2 cells (Figure 2A). Cisplatin (20 μM) exposure caused a prominent cell viability loss, while gradient ECH pretreatment restored cell viability in a concentration-dependent manner (Figure 2B).

PI staining was used to quantify dead tubular epithelial cells. Confocal images showed extensive PI-positive staining in cisplatin-stimulated HK-2 cells, and ECH co-treatment significantly reduced the proportion of PI-positive cells (Figure 2C). Western blot results showed that cisplatin dramatically increased KIM-1 and NGAL protein levels, which were reduced by ECH supplementation (Figure 2D). In accordance with the in vivo findings, qRT-PCR data illustrated that cisplatin markedly upregulated the mRNA levels of IL-1β, MCP-1, TNF-α, and IL-6, and ECH intervention suppressed the overexpression of these inflammatory transcripts (Figure 2E–H). These cellular results confirmed that ECH directly alleviated cisplatin-induced HK-2 cell injury and inflammatory activation.

### 3.3. Single-Cell Transcriptomics and 4D-DIA Proteomics Point to Ferroptosis as a Candidate Pathogenic Pathway in Injured Proximal Tubular Epithelial Cells

**Figure 3:**
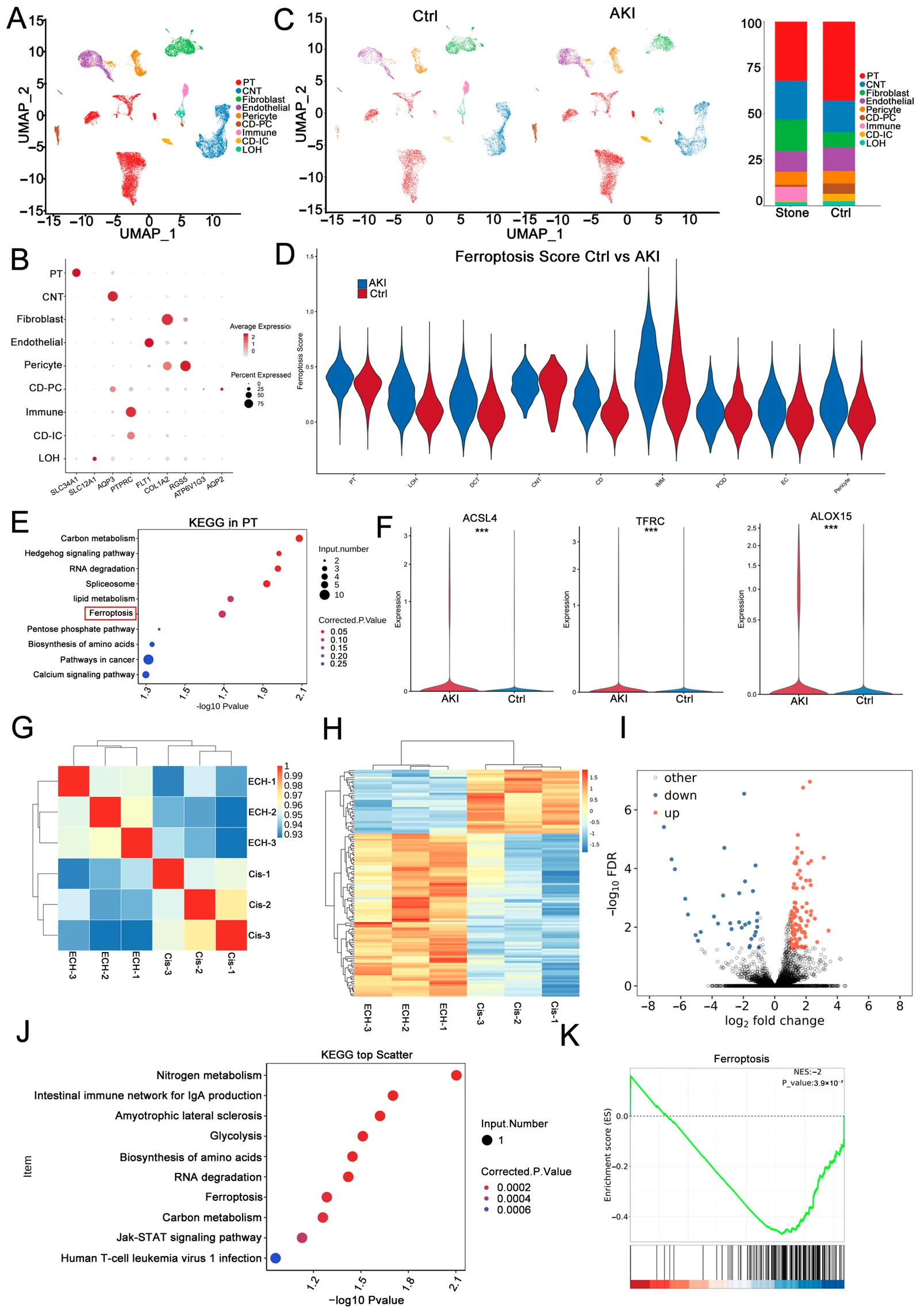
Single-cell transcriptomics and 4D-DIA proteomics identify ferroptosis as a candidate injury pathway in proximal tubules. (**A**) UMAP plot of renal cell subtypes. (**B**) Dot plot of cell marker genes. (**C**) UMAP and cell proportion statistics of control and AKI groups. (**D**) Violin plots of ferroptosis scores across cell clusters. (**E**) KEGG enrichment scatter plot of differentially expressed genes in proximal tubules. (**F**) Violin plots of ACSL4, TFRC and ALOX15 expression. (**G**) Correlation heatmap of 4D-DIA proteomic samples. (**H**) Clustering heatmap of differentially expressed proteins. (**I**) Volcano plot of differentially expressed proteins. (**J**) Scatter plot of top enriched KEGG pathways. (**K**) GSEA curve of ferroptosis pathway at protein level. *** *p* < 0.001 vs. control group.

To clarify the key pathological cascade and critical cell population involved in cisplatin-induced renal damage, we first analyzed public single-cell RNA-seq datasets from cisplatin-injured mouse kidneys. UMAP dimensionality reduction separated renal cells into distinct subtypes annotated by canonical marker genes, among which proximal tubular epithelial cells constituted the primary damaged cell population after cisplatin stimulation (Figure 3A–C). Calculation of ferroptosis signature scores across all cell clusters revealed that proximal tubule cells exhibited the highest ferroptosis score in the AKI group (Figure 3D). KEGG enrichment analysis of differentially expressed genes within proximal tubular cells highlighted ferroptosis as a significantly enriched signaling pathway (adjusted *p* = 0.000265, Figure 3E). Compared with other cell-death-related pathways, apoptosis and necroptosis displayed relatively weaker enrichment signals in this analysis. Violin plots further validated that the ferroptosis-associated genes ACSL4, ALOX15, and the iron uptake gene TFRC were all significantly upregulated in cisplatin-damaged proximal tubular cells (Figure 3F).

We further performed 4D-DIA quantitative proteomics with ion mobility diaPASEF separation to compare global protein profiles of renal tissues from the cisplatin model and ECH intervention groups [30,31]. The sample correlation heatmap verified reliable reproducibility across biological replicates (Figure 3G). The volcano plot and hierarchical clustering heatmap visualized extensive protein expression alterations induced by cisplatin, which were partially reversed following ECH treatment (Figure 3H,I). GSEA results confirmed significant enrichment of the ferroptosis pathway at the protein level in cisplatin-challenged kidneys, whereas ECH intervention effectively restrained ferroptosis pathway activation (Figure 3J,K). Collectively, the integrated analysis of single-cell transcriptome and proteomic data indicated that ferroptosis served as an important pathological event mediating cisplatin-triggered tubular injury, and ECH inhibited ferroptosis at both the transcriptional and protein levels.

### 3.4. ECH Restores Slc7a11/GPX4 Antioxidant Axis to Suppress Lipid Peroxidation and Ferroptosis in HK-2 Cells

**Figure 4:**
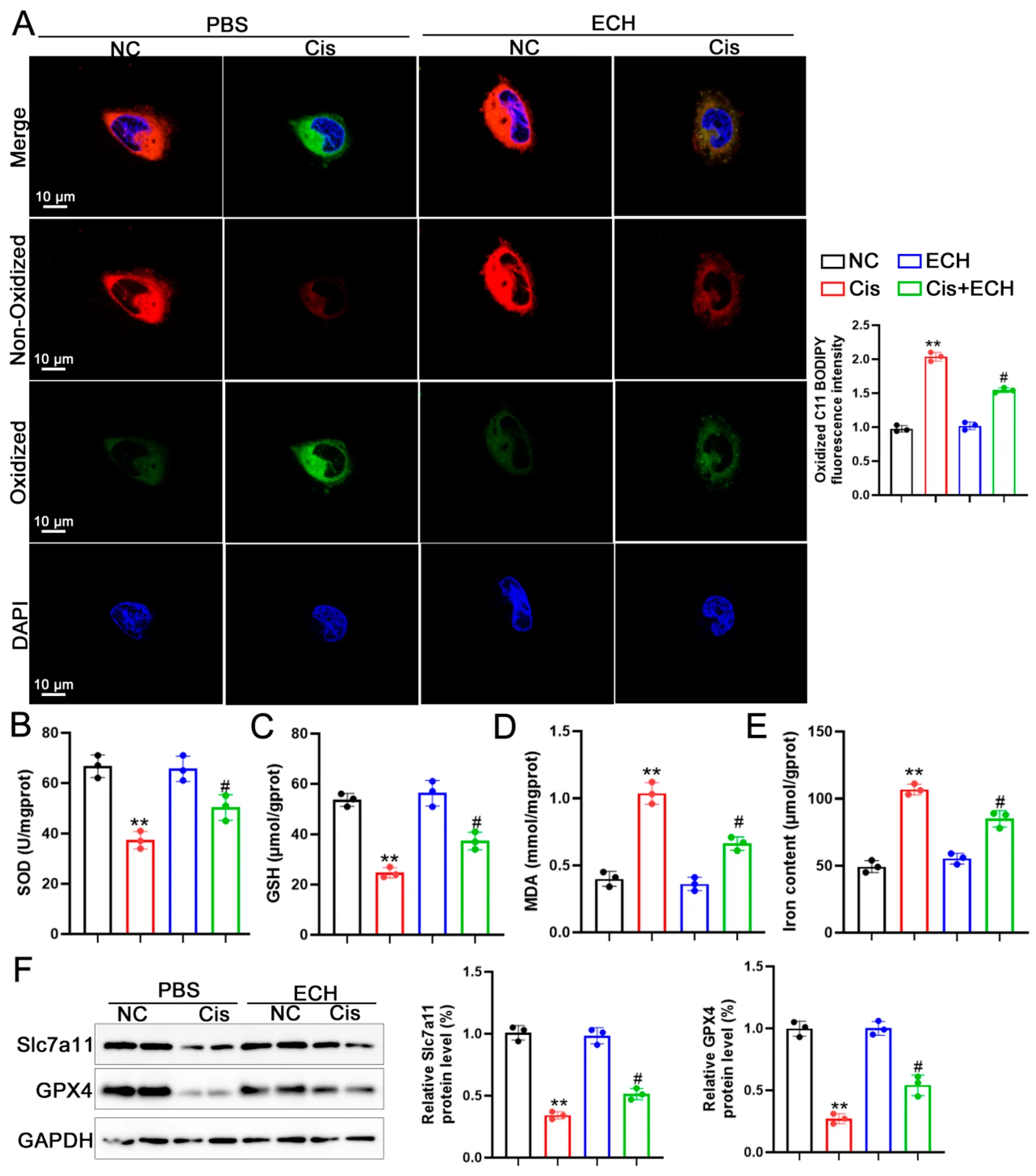
ECH restores Slc7a11/GPX4 axis to suppress ferroptosis in vitro. (**A**) Representative C11 BODIPY staining images of HK-2 cells and quantitative analysis of oxidized lipid fluorescence intensity. (**B**) SOD levels in HK-2 cells. (**C**) GSH levels in HK-2 cells. (**D**) MDA levels in HK-2 cells. (**E**) Iron content in HK-2 cells. (**F**) Western blot analysis of Slc7a11 and GPX4 proteins in HK-2 cells. ** *p* < 0.01 vs. control group; ^#^ *p* < 0.05 vs. cisplatin group.

The C11-BODIPY fluorescent probe was applied to detect intracellular lipid peroxidation. Confocal imaging showed intense green oxidized lipid fluorescence in cisplatin-treated HK-2 cells, and ECH pretreatment largely eliminated lipid peroxide accumulation (Figure 4A). Detection of cellular redox indicators revealed that cisplatin induced decreased SOD and GSH levels and elevated MDA and ferrous iron levels, whereas ECH significantly recovered antioxidant capacity and relieved iron overload (Figure 4B–E).

Western blot analysis was used to detect core ferroptosis defense proteins. Cisplatin exposure led to notable downregulation of Slc7a11 and GPX4, two critical components of the ferroptosis inhibitory axis. ECH dose-dependently restored the expression of Slc7a11 and GPX4 proteins (Figure 4F). These cellular biochemical and molecular results demonstrated that ECH inhibited cisplatin-induced ferroptosis by rescuing the impaired Slc7a11/GPX4 antioxidant pathway.

### 3.5. ECH Alleviates Mitochondrial Ultrastructural Damage and Ferroptotic Redox Disturbance in Cisplatin-Injured Kidney Tissues

**Figure 5:**
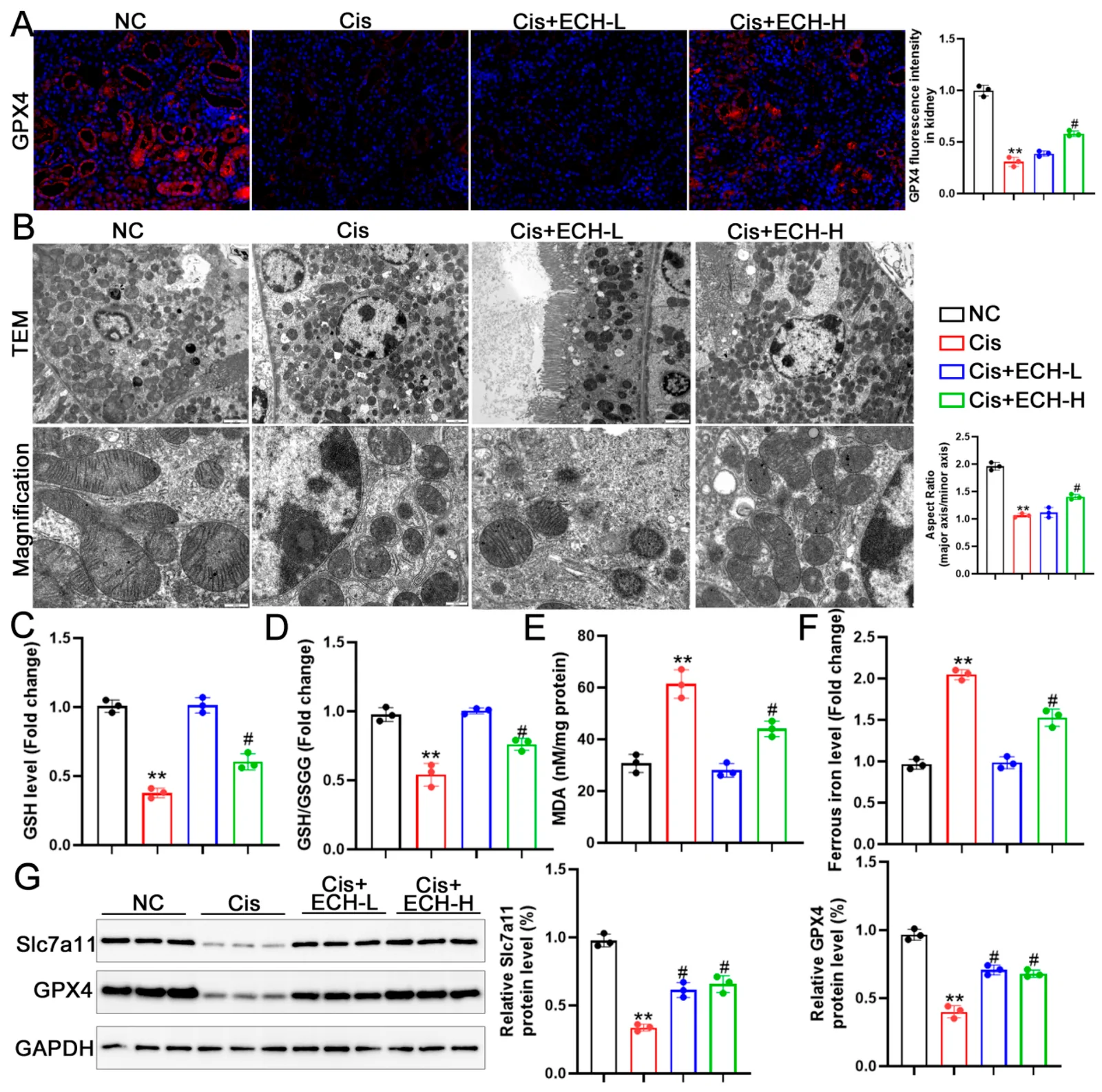
ECH attenuates mitochondrial ferroptotic damage in vivo. (**A**) Representative GPX4 immunofluorescence staining images of kidney tissues and quantitative analysis of fluorescence intensity. (**B**) Representative TEM images of renal tubular mitochondria and quantitative analysis of mitochondrial aspect ratio. (**C**) GSH levels in kidney tissues. (**D**) GSH/GSSG ratio in kidney tissues. (**E**) MDA levels in kidney tissues. (**F**) Ferrous iron levels in kidney tissues. (**G**) Western blot analysis of Slc7a11 and GPX4 proteins in kidney tissues. ** *p* < 0.01 vs. control group; ^#^ *p* < 0.05 vs. cisplatin group. *n* = 3 mice per group.

GPX4 immunofluorescence staining was performed to assess renal ferroptosis defense status in vivo. The cisplatin group exhibited drastically weakened GPX4 fluorescence intensity in renal tubules, while both low and high doses of ECH obviously enhanced GPX4 fluorescent signals (Figure 5A). Transmission electron microscopy (TEM) was adopted to observe mitochondrial morphological changes, a hallmark of ferroptosis. Tubular mitochondria in cisplatin-treated mice displayed severe swelling, fragmentation, and cristae dissolution, accompanied by a decreased mitochondrial aspect ratio. ECH treatment restored normal tubular mitochondrial morphology and elevated the mitochondrial aspect ratio (Figure 5B).

Consistent with the cellular test results, tissue biochemical analysis showed reduced GSH content, a decreased GSH/GSSG ratio, and increased MDA and ferrous iron deposition in cisplatin-treated renal tissues, all of which were reversed by ECH administration (Figure 5C–F). Western blot quantification further validated that ECH dose-dependently rescued the protein expression of Slc7a11 and GPX4 in cisplatin-damaged kidney tissues (Figure 5G). In vivo phenotypic data confirmed that ECH mitigated mitochondrial ferroptotic injury and restored tissue antioxidant homeostasis via activating the Slc7a11/GPX4 axis.

### 3.6. Molecular Docking and Molecular Dynamics Simulation Predict a Potential Interaction Between ECH and Bap1

**Figure 6:**
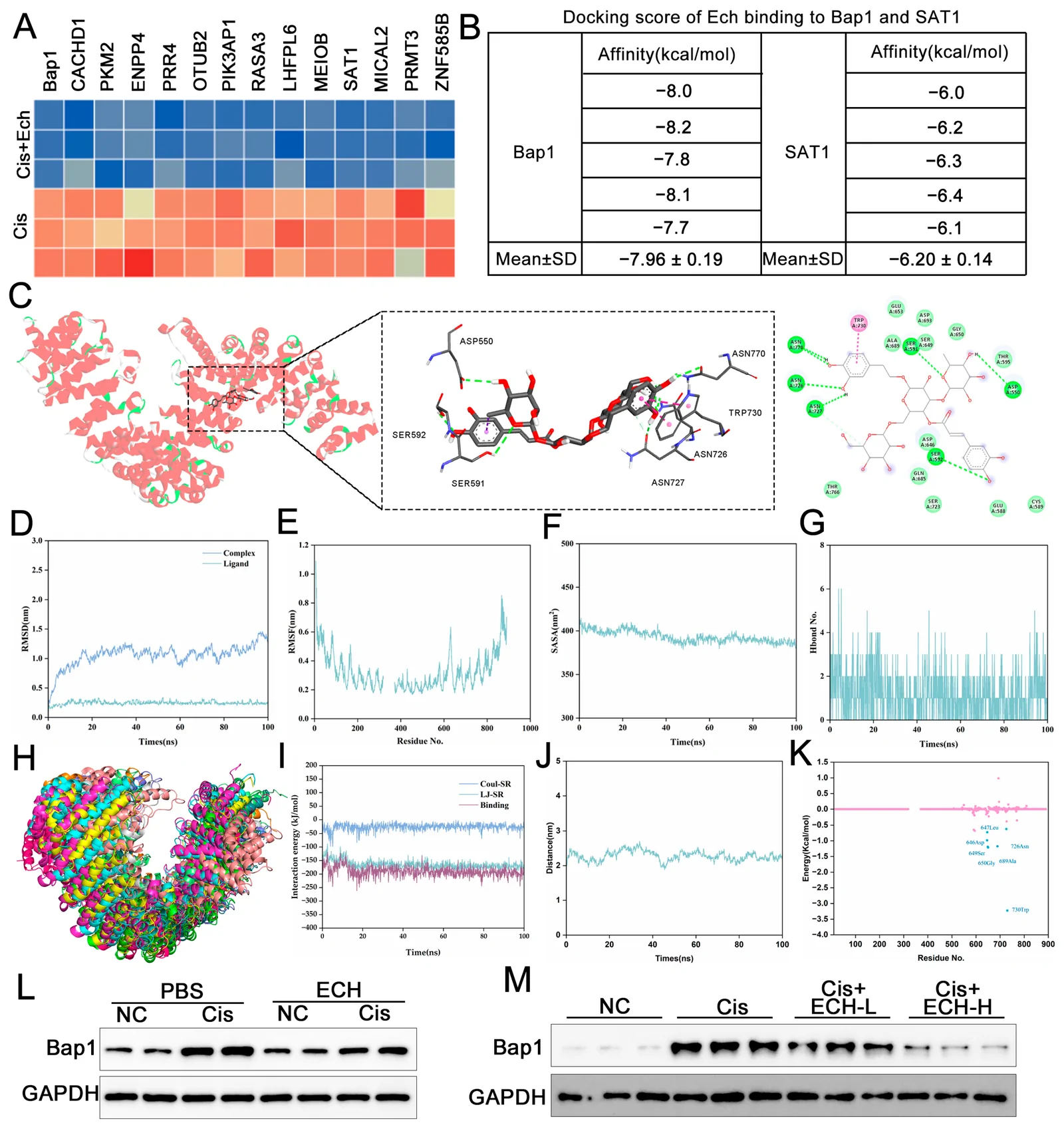
ECH potentially interacts with Bap1 and reduces Bap1 expression. (**A**) Heatmap of ferroptosis-associated proteins from 4D-DIA proteomic data. (**B**) Binding affinity scores of ECH with Bap1 and SAT1. (**C**) 3D docking structure and 2D residue interaction diagram of ECH-Bap1 complex. (**D**–**K**) Molecular dynamics curves of ECH-Bap1 complex, including RMSD, RMSF, SASA, hydrogen bond, trajectory conformation, interaction energy, ligand distance and residue binding free energy. (**L**) Western blot of Bap1 protein in HK-2 cells. (**M**) Western blot analysis of Bap1 protein in kidney tissues.

We used 4D-DIA quantitative proteomics to screen differently expressed proteins in renal tissues from the Cis and Cis + ECH groups. Global DEPs were intersected with ferroptosis-associated protein sets to obtain candidate molecules. Among these candidates, Bap1 was prioritized owing to its significant protein-level alteration, documented ferroptosis-regulating function, and pathological relevance to kidney injury. The heatmap illustrated obvious upregulation of Bap1 upon cisplatin stimulation (Figure 6A). To predict the potential interaction of ECH with Bap1 and the reference protein SAT1, molecular docking analysis was performed. The average binding free energy of the ECH-BAP1 complex was −7.96 ± 0.19 kcal/mol, which was substantially lower than that of the ECH-SAT1 complex (−6.20 ± 0.14 kcal/mol), indicating higher predicted binding affinity of ECH toward BAP1 (Figure 6B). These affinity metrics were obtained from multiple computational docking conformations. The optimal docking conformation showed that ECH inserted into the hydrophobic pocket of Bap1 and formed multiple hydrogen bonds and hydrophobic interactions with key amino acid residues (Figure 6C).

A 100 ns all-atom molecular dynamics simulation was further performed to assess the dynamic stability of the ECH-Bap1 complex. RMSD curves of the complex and free ligand reached equilibrium after 20 ns of simulation, suggesting that the overall conformation of the binding system remained stable without dramatic structural shifts (Figure 6D). RMSF analysis indicated that residues within the ligand-binding pocket of Bap1 exhibited low fluctuation, supporting the rigid nature of the binding cavity (Figure 6E). The solvent-accessible surface area (SASA) of the complex maintained a relatively steady level throughout the simulation process (Figure 6F). The number of intermolecular hydrogen bonds between ECH and Bap1 fluctuated between zero and six, sustaining continuous intermolecular interactions (Figure 6G). Superimposed trajectory snapshots visually verified that ECH was steadily trapped inside the pocket and did not dissociate during the entire simulation (Figure 6H). The total intermolecular interaction energy stayed at a constant low level without obvious upward drift (Figure 6I). The centroid distance between the ECH ligand and the Bap1 binding pocket remained stable, showing no separation trend (Figure 6J). Residue-based binding free energy decomposition further identified core amino acid residues that dominated the binding force of the ECH-Bap1 complex (Figure 6K).

Western blot assays were next conducted to detect the regulatory effect of ECH on Bap1 expression at the cellular and in vivo levels. In HK-2 tubular epithelial cells, cisplatin treatment markedly elevated Bap1 protein abundance, while ECH co-treatment reversed this upregulation (Figure 6L). Consistently, renal Bap1 expression was significantly increased in cisplatin-injured mice, and both low-dose and high-dose ECH intervention effectively reduced renal Bap1 protein levels (Figure 6M). Collectively, molecular docking and the 100 ns molecular dynamics simulation predicted a potential interaction between ECH and Bap1, and Western blot results demonstrated that ECH suppressed Bap1 expression both in vitro and in vivo.

### 3.7. Overexpression of Bap1 Largely Abrogates ECH-Mediated Anti-Ferroptosis and Renoprotective Effects

**Figure 7:**
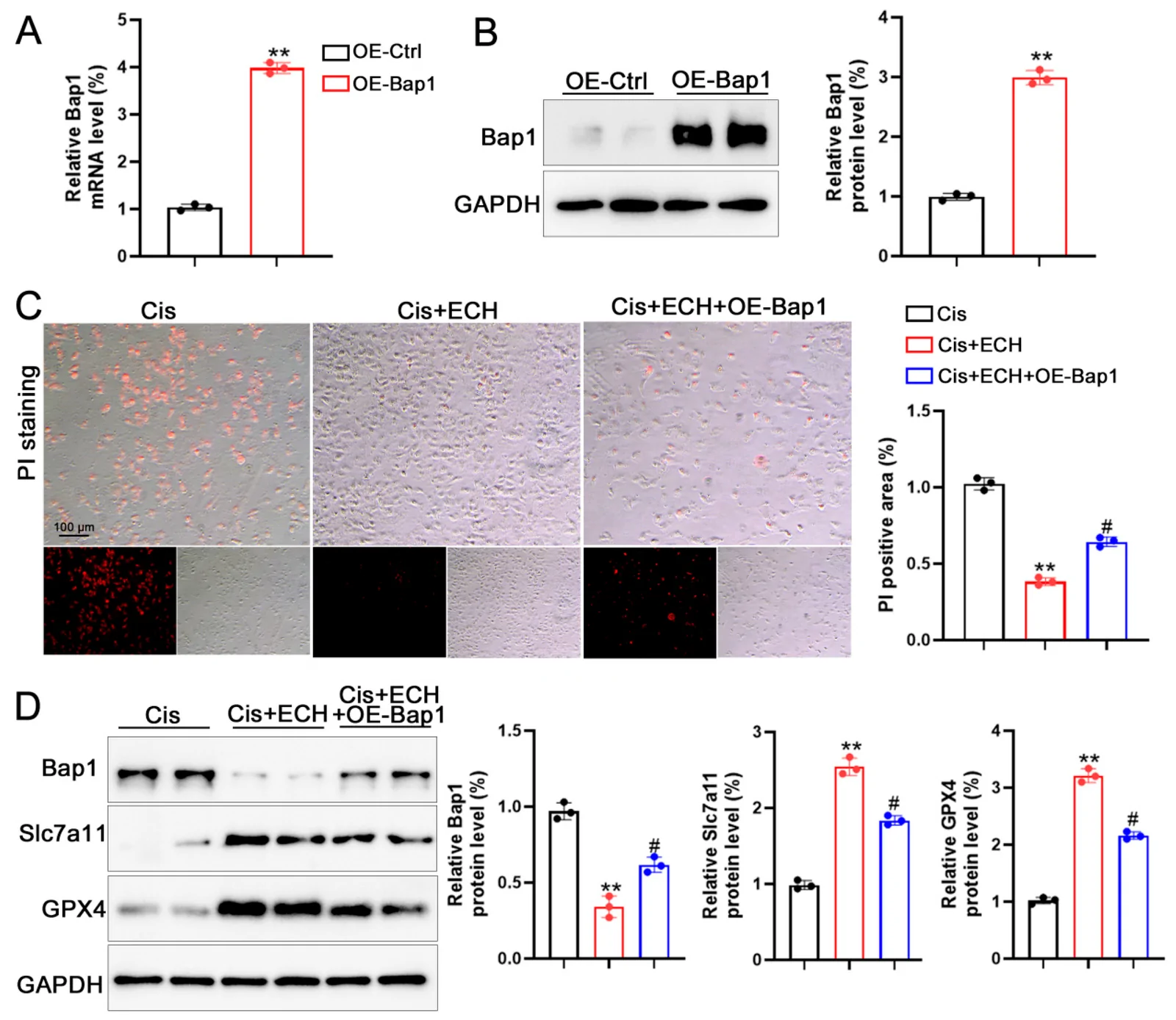
Overexpression of Bap1 largely abrogates renoprotective effects of ECH. (**A**) Relative Bap1 mRNA levels in HK-2 cells. (**B**) Western blot and quantitative analysis of Bap1 protein in HK-2 cells. (**C**) Representative PI staining images of HK-2 cells and quantitative analysis of the PI-positive area. (**D**) Western blot analysis of Bap1, Slc7a11 and GPX4 proteins in HK-2 cells. ** *p* < 0.01 vs. OE-Ctrl group (**A**,**B**) or cisplatin group (**C**,**D**); ^#^ *p* < 0.05 vs. Cisplatin + ECH group.

Rescue experiments with a Bap1 overexpression plasmid were conducted to validate whether Bap1 mediates the renoprotective function of ECH. qRT-PCR and Western blot analysis verified successful overexpression of the Bap1 in HK-2 cells transfected with Bap1 overexpression vector (Figure 7A,B). PI staining results showed that ECH efficiently reduced cisplatin-triggered cell death, whereas Bap1 overexpression largely abrogated the cytoprotective effect of ECH (Figure 7C). Western blot analysis of ferroptosis markers revealed that ECH upregulated Slc7a11 and GPX4 proteins and suppressed Bap1 expression under cisplatin stimulation. However, forced Bap1 overexpression markedly counteracted ECH’s regulatory effects on Slc7a11 and GPX4, and restored elevated Bap1 abundance (Figure 7D). These rescue assays demonstrated that Bap1 downregulation contributes substantially to ECH-mediated anti-ferroptotic and tubular-protective effects.

## 4. Discussion

Cisplatin-induced acute kidney injury severely restricts the clinical application of this widely used chemotherapeutic agent, and effective targeted protective interventions remain absent. In the present study, multi-omics data collectively point to ferroptosis as a candidate pathological program activated in cisplatin-injured renal proximal tubules. Consistent with published evidence, our phenotypic assays indicate that ferroptosis-related lipid peroxidation and mitochondrial damage are prominent pathological features in cisplatin-damaged tubular epithelium, and ECH treatment mitigates these ferroptotic alterations both in vivo and in vitro.

Ferroptosis is driven by iron-dependent lipid peroxidation, and the Slc7a11/GPX4 axis serves as a central defensive module limiting this form of cell death. In our dataset, cisplatin suppressed Slc7a11/GPX4 signaling, whereas ECH restored this antioxidant cascade to counteract lipid peroxidation. These observations align with existing literature documenting disrupted Slc7a11/GPX4 activity in cisplatin-triggered AKI and underscore the therapeutic potential of restoring this axis for tubular protection.

In our model, ECH suppresses ferroptosis at both the lipid-peroxidation and mitochondrial-damage levels. C11-BODIPY staining demonstrated that ECH markedly reduced intracellular lipid peroxide accumulation in cisplatin-treated HK-2 cells, while transmission electron microscopy revealed that ECH restored mitochondrial morphology—including reduced swelling, preserved cristae structure, and elevated mitochondrial aspect ratio—in cisplatin-injured renal tubules. These two readouts are mechanistically linked: lipid peroxidation is a defining biochemical hallmark of ferroptosis, whereas mitochondrial structural deterioration represents a characteristic ultrastructural manifestation of ferroptotic cell death. Our findings are consistent with the NRF2/GPX4–ferroptosis–mitochondrial injury axis previously reported for licochalcone A in cisplatin nephrotoxicity [32].

We further explored the upstream target through 4D-DIA proteomic screening and molecular verification. Consistent with earlier reports describing Bap1 as a catalytic subunit within the PR-DUB complex that mediates epigenetic repression of Slc7a11 transcription, Bap1 was found to be significantly elevated at the protein level in cisplatin-damaged renal tissue. Molecular docking and a 100 ns molecular dynamics simulation predicted a potential interaction between ECH and Bap1, with continuous hydrogen-bond interactions observed throughout the simulation trajectory. Western blot assays further validated that ECH dose-dependently downregulated Bap1 protein expression in both HK-2 cells and mouse kidneys. More importantly, Bap1 overexpression largely abrogated the anti-ferroptotic and cytoprotective functions of ECH, indicating that Bap1 inhibition contributes substantially to ECH-conferred renoprotection. To date, no natural small molecule has been reported to modulate Bap1 to relieve cisplatin nephrotoxicity, which represents the major innovation of this work.

Numerous studies have confirmed the anti-inflammatory and antioxidant properties of ECH in multiple organ injury models. Our research expands the functional spectrum of ECH by establishing a novel regulatory relationship: ECH downregulates Bap1 protein abundance, which is accompanied by restoration of the Slc7a11/GPX4 axis and suppression of ferroptosis.

Nevertheless, the mechanistic link between the predicted potential ECH-Bap1 interaction and the reduction in Bap1 protein abundance remains incompletely resolved. Two plausible scenarios could explain this observation. On the one hand, if ECH physically binds to Bap1, ligand occupancy may alter the Bap1 conformational state, which could trigger post-translational modification and subsequent protein degradation. On the other hand, ECH may suppress Bap1 expression through transcriptional or post-transcriptional indirect effects independent of direct protein–ligand contact. Since we did not perform assays for Bap1 mRNA levels, ubiquitination, or protein turnover in the present study, we cannot distinguish these possibilities, which represents an important unresolved mechanistic question for future work.

Prior literature has well characterized the classical antioxidant actions of ECH, including direct ROS scavenging and activation of NRF2-mediated antioxidant responses. Our study identifies a Bap1-Slc7a11/GPX4-dependent ferroptosis regulatory axis as an additional protective mechanism. We propose that this newly described pathway operates in parallel with, rather than replacing, previously reported antioxidant circuits of ECH. Consistent with this interpretation, Bap1 overexpression largely attenuates but does not completely abrogate ECH-mediated cytoprotection, indicating that ECH renoprotection is partially dependent on Bap1 and that other antioxidant pathways still contribute to its overall beneficial effect.

From a clinical translational perspective, several considerations warrant discussion. Currently, no standard renoprotective drug is routinely recommended for all cisplatin-treated patients. Amifostine, the only FDA-approved agent for reducing cumulative cisplatin renal toxicity in advanced ovarian cancer, is limited by side effects, including hypotension and nausea, as well as intravenous-only administration, restricting its broad application [33]. A critical concern for any systemic cytoprotective agent is the theoretical risk of protecting tumor cells and thereby compromising chemotherapy efficacy. Our study was conducted in a non-tumor-bearing AKI model with preventive ECH pretreatment, and we did not evaluate whether ECH affects the anti-tumor activity of cisplatin; tumor-bearing models are therefore required to exclude this potential risk. As a natural compound with a favorable safety profile and potential oral bioavailability, ECH may offer practical advantages over amifostine, although direct comparative studies are needed. Finally, both ECH and cisplatin doses would require careful optimization in clinical translation; whether our findings extend to clinical cisplatin dosing regimens and whether ECH dose adjustment is needed remain open questions requiring pharmacokinetic and clinical investigation.

It is worth noting that the effect of ECH on ferroptosis appears to be context-dependent. While ECH suppresses ferroptosis in steatotic liver and cisplatin-induced AKI models, it has been reported to promote ferroptosis in hepatocellular carcinoma cells [34,35,36]. This dual role may arise from differences in cell type, baseline metabolic state, drug dosage, and disease microenvironment. In our cisplatin-injured renal tubular model, our phenotypic and biochemical data consistently demonstrate that ECH acts as a ferroptosis suppressor.

Nevertheless, several limitations of this study need to be addressed. First, all in vivo ECH treatments in our animal model were given as preventive prophylactic pretreatment before cisplatin insult, rather than as a therapeutic intervention for established kidney injury. Therefore, our findings demonstrate preventive renoprotection and cannot directly support therapeutic efficacy against established cisplatin-induced AKI. Additional animal experiments with post-damage drug administration are required to better reflect real-world clinical scenarios. Second, we only performed Bap1 overexpression rescue assays in HK-2 cells, and tubule-specific Bap1 knockout mouse models were not applied to further confirm the causal relationship between Bap1 and ECH-mediated renoprotection. Third, this work solely focused on the renoprotective activity of ECH without exploring whether ECH would compromise the anti-tumor efficacy of cisplatin; tumor-bearing animal models are needed to exclude potential risks of weakened chemotherapy effects. Fourth, molecular docking and molecular dynamics simulations in the present study only predicted a plausible interaction between ECH and Bap1. Definitive biochemical target engagement assays such as SPR, MST, or a cellular thermal shift assay are needed in future investigations to experimentally verify their direct physical binding. In addition, the intermediate mechanism linking this predicted interaction to reduced Bap1 protein abundance remains to be further dissected. Only a single 100 ns molecular dynamics trajectory with one random seed was generated for the ECH-Bap1 complex without independent replicate simulations using different random seeds; multiple replicate simulations are required in future work to achieve more adequate conformational sampling. Fifth, pharmacological rescue experiments using canonical ferroptosis inhibitors (e.g., ferrostatin-1 or liproxstatin-1) were not performed in the current study to pharmacologically dissect the functional contribution of ferroptosis inhibition. Further experiments incorporating these inhibitors will be carried out in future investigations. Sixth, only male mice were used for all in vivo experiments in this study. Considering well-established sex-dependent differences in cisplatin-provoked nephrotoxicity and ferroptosis susceptibility, our current observations may not be fully generalizable to female animals. Future studies involving both male and female mice are required to evaluate potential sex-related disparities. Key in vivo functional experiments were performed with *n* = 3 biological replicates per group. While consistent phenotypic trends were observed across replicates, this relatively small sample size may limit the statistical robustness and broad generalizability of our in vivo findings. Larger animal cohorts would be beneficial for further validation in future investigations. Furthermore, although prior studies have demonstrated Bap1-mediated repression of Slc7a11 transcription, we did not perform ChIP-seq or luciferase reporter assays to verify direct promoter-binding events in our cisplatin-injured kidney model. Further experiments are required to dissect this regulatory mechanism. Subsequent research will construct post-injury treatment models and Bap1 conditional knockout mice, as well as tumor-bearing models, to resolve the above deficiencies.

In summary, this work clarifies a unique molecular mechanism by which ECH confers preventive renoprotection against cisplatin-induced acute kidney injury by downregulating Bap1 to inhibit tubular ferroptosis. The ECH/Bap1/ferroptosis regulatory relationship provides a novel theoretical basis and potential therapeutic target for the clinical prevention of cisplatin-associated nephrotoxicity, and ECH is expected to serve as a safe natural adjuvant agent during cisplatin chemotherapy.

## Data Availability

The data presented in this study are available on request from the corresponding author due to ethical reasons.
